# Transcriptomic analysis delineates preterm prelabor rupture of membranes from preterm labor in preterm fetal membranes

**DOI:** 10.1186/s12920-024-01841-7

**Published:** 2024-03-05

**Authors:** Lori A. Underhill, J. M. Mennella, G. A. Tollefson, A. Uzun, B. E. Lechner

**Affiliations:** 1 Warren Alpert Medical School at Brown University, Providence, RI USA; 2 Department of Pediatrics, Women and Infants Hospital, Providence, RI USA; 3 Women and Infants Hospital, 101 Dudley St, 02905 Providence, RI USA

**Keywords:** RNA-Seq, Preterm birth, Preterm prelabor rupture of membranes, Amnion, Chorion

## Abstract

**Background:**

Globally, preterm birth remains the leading cause of death in children younger than 5 years old. Spontaneous preterm birth is comprised of two events that may or may not occur simultaneously: preterm labor and preterm prelabor rupture of membranes (PPROM). To further explore the concept that spontaneous preterm birth can result from the initializing of two separate but overlapping pathological events, we compared fetal membrane tissue from preterm labor deliveries to fetal tissue from preterm labor with PPROM deliveries. We hypothesized that the fetal membrane tissue from preterm labor with PPROM cases will have an RNA-seq profile divergent from the fetal membrane tissue from preterm labor controls.

**Methods:**

Chorioamnion, separated into amnion and chorion, was collected from eight gestationally age-matched cases and controls within 15 min of birth, and analyzed using RNA sequencing. Pathway enrichment analyses and functional annotations of differentially expressed genes were performed using KEGG and Gene Ontogeny Pathway enrichment analyses.

**Results:**

A total of 1466 genes were differentially expressed in the amnion, and 484 genes were differentially expressed in the chorion (log2 fold change > 1, FDR < 0.05) in cases (preterm labor with PPROM), versus controls (preterm labor only). In the amnion, the most significantly enriched (FDR < 0.01) KEGG pathway among down-regulated genes was the extracellular matrix receptor interaction pathway. Seven of the most significantly enriched pathways were comprised of multiple genes from the COL family, including COL1A, COL3A1, COL4A4, and COL4A6. In the chorion, the most significantly enriched KEGG pathways in up-regulated genes were chemokine, NOD receptor, Toll-like receptor, and cytokine-cytokine receptor signaling pathways. Similarly, KEGG pathway enrichment analysis for up-regulated genes in the amnion included three inflammatory pathways: cytokine-cytokine interaction, TNF signaling and the CXCL family. Six genes were significantly up regulated in chorionic tissue discriminated between cases (preterm labor with PPROM) and controls (preterm labor only) including GBP5, CXCL9, ALPL, S100A8, CASP5 and MMP25.

**Conclusions:**

In our study, transcriptome analysis of preterm fetal membranes revealed distinct differentially expressed genes for PPROM, separate from preterm labor. This study is the first to report transcriptome data that reflects the individual pathophysiology of amnion and chorion tissue from PPROM deliveries.

**Supplementary Information:**

The online version contains supplementary material available at 10.1186/s12920-024-01841-7.

## Background

Preterm birth remains the leading cause of death in children younger than 5 years old and is defined by the World Health organization as births before 37 completed weeks of gestation or fewer than 259 days from the first date of a woman’s last menstrual period [1]. Most recent global estimates show approximately 10.6% of all live births are preterm, while in the U.S., the rate is 10.1% [2].

Multiple preterm birth classifications have been created to help guide research on risk determinants, pathology, and epidemiology of this syndrome. The etiology of preterm birth is multifactorial, with nutritional, environmental and socio-demographics all contributing to an increase in risk of occurrence [3]. It is believed that approximately 30% of preterm birth cases are caused by infection and inflammation [4]. More recently, it has been hypothesized that maternal vaginal, cervical, and intestinal microbiomes may contribute to the etiology of preterm birth [5–7].

Preterm birth can be classified as either spontaneous (spontaneous preterm birth), or provider initiated by cesarean or labor induction [4]. Spontaneous preterm birth is comprised of two events that may or may not occur simultaneously: preterm labor (PTL) and preterm prelabor rupture of membranes (PPROM). PTL occurs when regular contractions result in the opening of the cervix after week 20 but before week 37 of pregnancy. Although both term and preterm labor present with clinical similarities such as uterine contraction, and cervical dilation, it has been proposed that preterm labor is not merely labor that starts before 37 weeks of gestation, but a syndrome caused by multiple pathologies [8, 9].

PPROM is the spontaneous rupture of fetal membranes before labor before the 37th week of gestation. Fetal membranes are attached to the placenta, and are composed of two layers: the amnion, the layer directly in contact with amnionic fluid, and the chorion which is attached to the maternal decidua [10]. It has been hypothesized that PPROM is a disease of the fetal membranes initiated, in part, by the dysregulation of inflammatory pathways [11–13]. Connective tissue disorders are also associated with weakened fetal membranes and an increased incident of PPROM [14, 15].

Many studies have focused on the developmental events that differentiate the onset of spontaneous preterm birth from the onset of PPROM, yet we are still unable to elucidate the pathways that differentiate the two pathophysiologies. To further explore the concept that spontaneous preterm birth can result from the initializing of two separate but overlapping pathological events (PTL +/- PPROM), we compared fetal membrane tissue from PTL deliveries versus tissue from PTL w/ PPROM deliveries. In this study, we hypothesize that the fetal membrane tissue from PTL + PPROM cases will have an RNA-seq profile divergent from the fetal membrane tissue from PTL only controls.

## Methods

### Tissue collection

Chorioamnion, was collected within 15 min of delivery from pregnancies complicated by preterm birth between the gestational ages of 27 to 34 weeks at Women and Infants Hospital, in Providence, RI from April 2018- February 2019. Pregnancies which included illicit drug use, IVF, or known congenital anomalies were excluded from the study. A 3 cm^2^ portion of the chorioamnionic membrane was taken from the place of rupture for the vaginal deliveries, and from the incision site for c-section deliveries. The chorioamnion membrane was then separated into amnion and chorion and immediately frozen in liquid nitrogen, or placed in RNAlater solution, and stored at -80 °C. Data obtained from clinical records included vaginal or cesarean section delivery, gestational age at delivery, and chronological initiation of PPROM or preterm labor, maternal age, race/ethnicity, and parity (See Additional File 3). s Informed consent for the study has been waived by our local ethics committee, The Care New England Institutional Review Board.The tissue being collected is considered residual pregnancy tissue; thus informed consent was not necessary.For this study, gestational age, and delivery type (vaginal or cesarean) were matched for 4 sets of cases and controls. Control samples were defined as those in which PTL presented first (PTL only), while cases were defined as those in which PPROM presented first, followed by PTL (PPROM + PTL). Both amnion and chorion samples were included in this study. The 16 tissue samples were shipped to BGI Americas (Hong Kong, China) for RNA extraction and analysis.

Total RNA was extracted from each sample using a phenol-chloroform extraction protocol to produce mRNA. Total concentration of RNA was evaluated on an Agilent Bioanalyzer 2100 (Santa Clara, CA). The RNA sequencing sample quality was determined by 28 S/18S and RIN (RNA Integrity Number) values. All 16 samples met the requirements of library construction and sequencing established by BGI.

### RNA-Seq and data analysis

RNA-Seq library construction was conducted by BGI America. Double stranded PCR products were circularized by the splint oligo sequence, and the resulting single strand circle DNA (ssCir DNA) was formatted as the final library. The library was amplified with phi29 to make DNA nanoball (DNB) which has more than 300 copies of one molecule. The DNBs were loaded into a patterned nanoarray and paired-end 100 base reads were generated by Probe-Anchor Synthesis (cPAS).

Raw fastq files for all samples were checked for quality using FastQC v0.11.5 (https://www.bioinformatics.babraham.ac.uk/projects/fastqc/) and did not require quality or adapter trimming. Salmon v1.3.0 [16] was then used in mapping-based mode to align reads and quantify transcripts. The Gencode v36 GRCh38.p13 human genome and Gencode v36 transcriptome versions were used for alignment. The R package txImport v1.22.0 [17] was used to import Salmon quant files containing transcript counts with bias correction data into R. Finally, the DESeq2 v1.31.6 R package [18] was used with default settings to perform differential expression analysis using the bias-corrected transcript counts from Salmon. Differential expression analysis was performed with three replicates per experiment group and compared PPROM unaffected versus affected tissue expression profiles in amnion and chorion tissue samples separately.

### Functional enrichment and annotation

We utilized the DAVID Database for Annotation, Visualization, and Integrated Discovery Functional Annotation Tool v6.8 [19, 20] to perform Gene Ontology (GO) and KEGG Pathway enrichment analyses on the genes which were differentially up-regulated or down-regulated by at least 2-fold. Genes with a p-value ≤ 0.05 and an absolute fold change ≥ 2 were significantly differentially expressed. GO terms and KEGG pathways were significantly enriched if they had a false discovery rate (FDR) corrected p-value ≤ 0.05.

Heatmaps of the DESeq2 functional enrichment results were prepared using the ggplot2 v3.3.6 R package [21].

### RT-PCR validation of RNASeq gene expression results

To confirm differentially expressed (DE) gene data, six genes were chosen for RT-PCR analysis based on a log2-fold changes higher than 4.0 and a highly statistically significant padj: guanylate binding protein 5 (GBP5), alkaline phosphatase, biomineralization associated (ALPL), S100 calcium binding protein A8 (S100A8), c-x-c motif chemokine ligand 9 (CXCL9), matrix metallopeptidase 25 (MMP25), and caspase 5 (CASP5). 8 cases and 8 controls, separate from the samples used for RNA-Seq, were analyzed. Each sample was assessed in duplicate, and the %CV between the duplicates was < 3%. All primers were ‘off the shelf’ sequences purchased from ThermoFisher Scientific ( Asheville, NC). The glyceraldehyde-3-phosphate dehydrogenase (GAPDH) gene was chosen as the reference genes to estimate relative quantification. The relative gene expression was calculated using the 2^−∆∆Ct^ method.”

The mean gestational age for both groups was 33.2 weeks Case and control data were compared using the Wilcoxon Rank sum test.

Total RNA was extracted from each sample using the RNeasy Plus mini kit (Qiagen, Germantown, MD), and the concentration and quality of the RNA was evaluated using a Nanodrop 2000 Spectrophotometer (ThermoFisher Scientific, Ashville, NC). cDNA was synthesized from the RNA using Superscript IV First-Strand Synthesis System (Thermo Scientific, Wilmington, DE)) using oligo primers, and performed in a SimpliAmp Thermal Cycler (Applied Biosystems, Asheville, NC) RT-PCR amplification was performed in Thermocycler 7500 using TaqMan Fast Universal Master Mix 2X Kit (ThermoFisher Scientific, Asheville, NC).

## Results

Demographic and clinical characteristics of the cases and control groups are presented in Table 1.

**Table 1:** Demographic and clinical characteristics of cases and controls for RNA-Seq

	Cases	Controls
	n=4	n=4
Maternal age + SD	32 + 6.8	27.75 + 5.3
Chorioamnionitis	0	0
Preeclampsia	1	0
Parity		
*nulliparous	1	1
>2	2	3

### Evaluation of gene expression

Genome-wide gene expression in amnion and chorion tissue were evaluated using RNA-Seq technology for 4 cases (PPROM + PTL) and 4 control (PTL only) samples. Cases and controls were matched by gestational age and type of delivery (vaginal or cesarean).

A summary of the read alignments to the reference genome is presented in Additional File 1. Sixteen samples were sequenced using BGISEQ platform, averaging about 4.66Gb bases per sample. The average mapping ratio with reference genome was 95.98%, the average mapping ratio with gene was 77.97%; 18,844 genes were identified.

A principal component analysis (PCA) was performed on the gene expression log-transformed values for the amnion and for the chorion, resulting in one case and one control being identified as outliers. PCA was rerun with 6 samples, resulting in a large portion of the variance being assigned to PC1 and PC2 in both the amnion (61%, 16% respectively) and the chorion (65%, 19% respectively). The PCA plot for the amnion showed equal variability between cases and controls, while the plot of the chorion samples showed a tight grouping of the controls, and large variability in the cases.

**Fig. 1:**
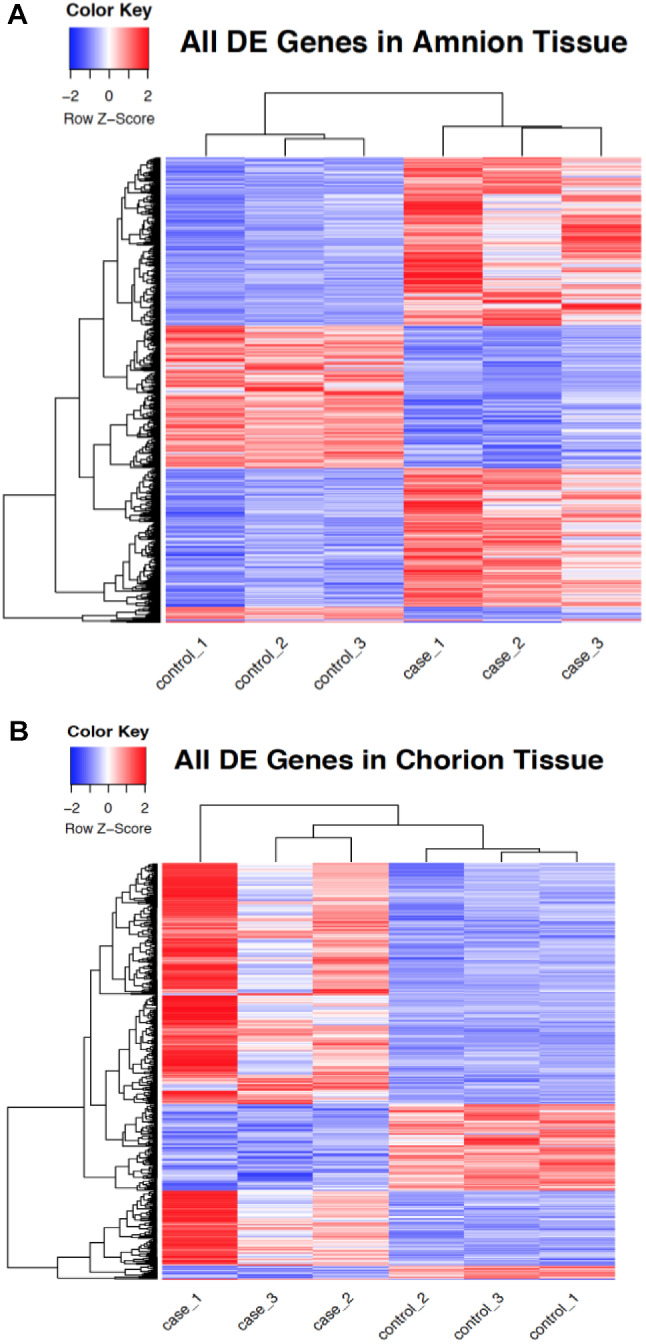
**A**. Heatmap of differentially expressed genes in PPROM affected amnion tissue. Heatmap of gene expression z-scores of 1466 differentially expressed genes clustered by row means between PPROM affected and unaffected amnion tissue. **B**. Heatmap of differentially expressed genes in PPROM affected chorion tissue. Heatmap of gene expression z-scores of 533 differentially expressed genes clustered by row means between PPROM affected and unaffected chorion tissue

A total of 1466 genes were differentially expressed (DE) in the amnion and 484 genes were differentially expressed in the chorion (log_2_fold change > 1, FDR < 0.05) (Fig. 1A, B). Among the 1466 DE genes in the amnion, 991 were upregulated and 475 were down regulated in cases versus controls (full list in Additional File 2a). Among the 484 DE genes in the chorion, 371 were upregulated and 113 were down regulated in cases versus controls (full list in Additional File 2b).

### Enrichment analysis using gene ontology

#### Amnion

**Fig. 2:**
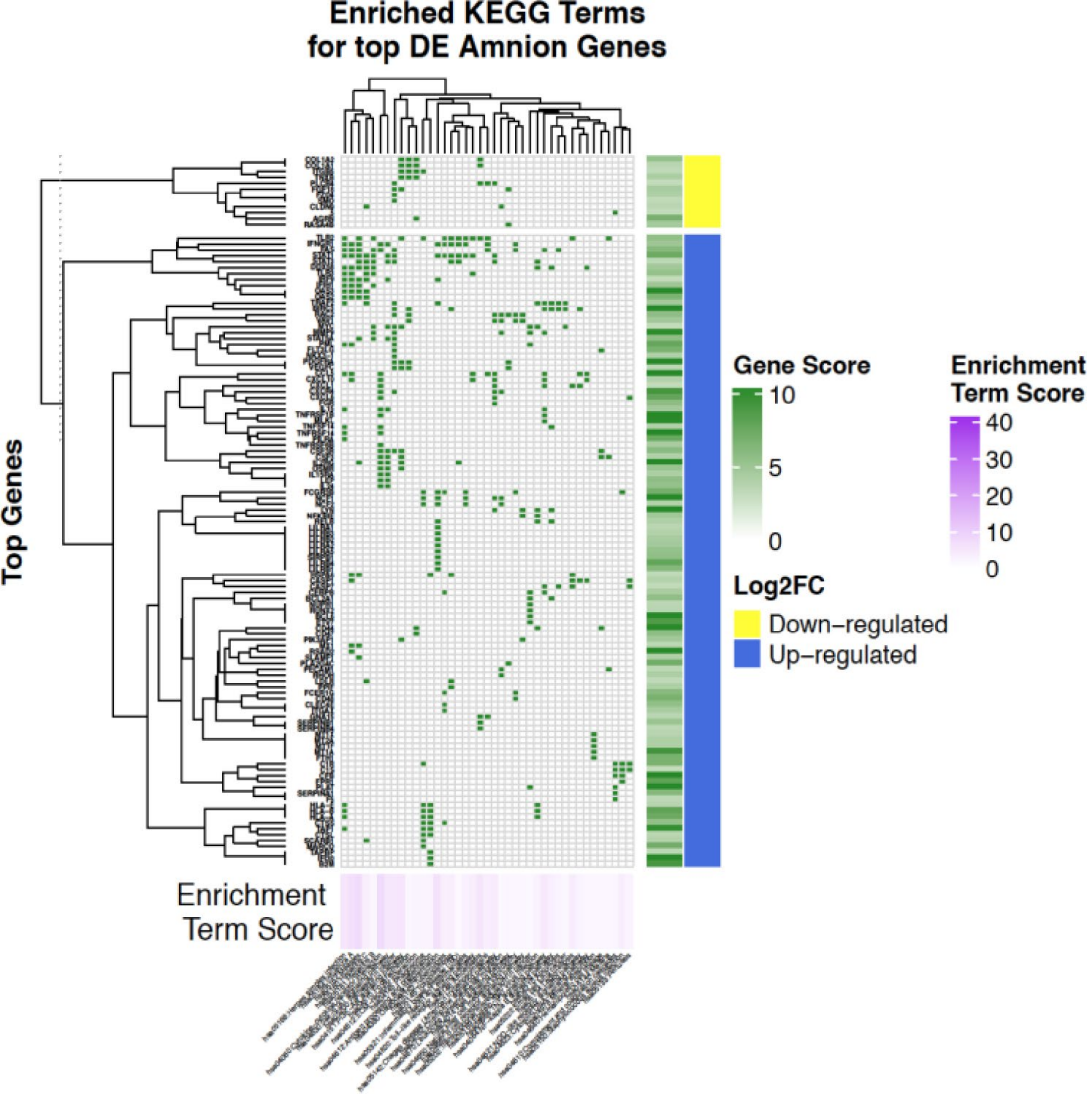
Clustered heatmap of significantly altered KEGG pathways in PPROM affected amnion tissue. Heatmap of differentially expressed genes between PPROM affected and unaffected amnion tissue among significantly enriched Kyoto Encyclopedia of Genes and Genomes (KEGG) pathways clustered by row and column means. The -log10(adjusted p-value) derived from differential expression analysis is illustrated by the Gene Score color bar. The false discovery rate (FDR)-corrected p-value derived from functional enrichment analysis using the DAVID Functional Annotation Tool is illustrated by the Enrichment Term Score color bar. The Log2FC color bar identifies sets of genes which are significantly up or downregulated. The significantly enriched KEGG pathways (FDR p-value < = 0.05) in which the differentially expressed genes are a member of are labeled on the lower axis

For KEGG pathways, the most significantly enriched (FDR < 0.01) pathway among the downregulated genes was the extracellular matrix (ECM) -receptor interaction pathway. Interestingly, 7 of the most significantly enriched pathways were comprised of multiple genes from the COL family, including COL1A1, COL3A1, COL4A4 and COL4A6 (Fig. 2). KEGG pathways that were significantly enriched (FDR < 0.01) for upregulated genes included the cytokine-cytokine receptor interaction pathway, TNF signaling pathway, and the CXCL family (Fig. 2).

**Fig. 3:**
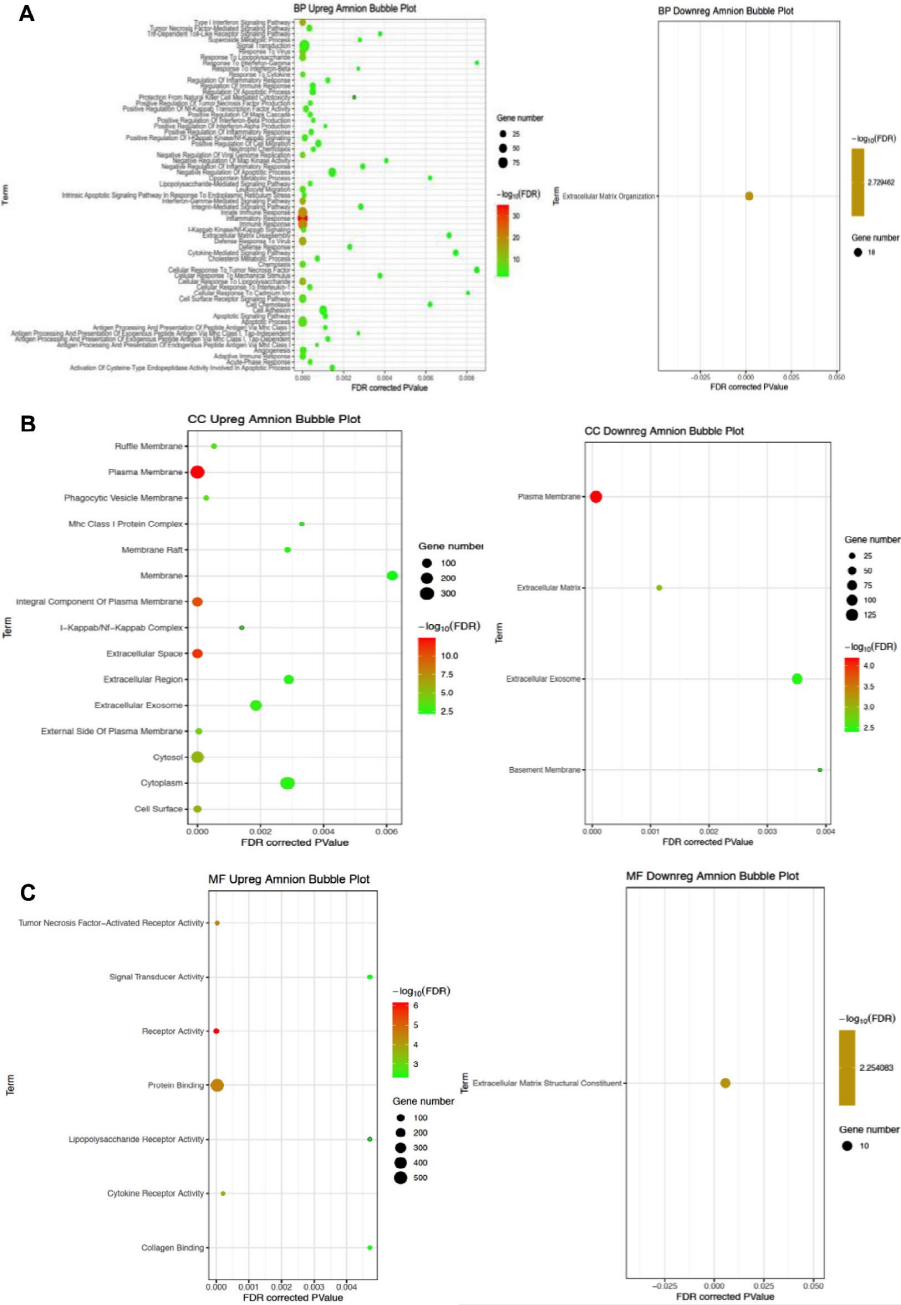
**A**. Bubble plots of significantly altered biological process gene ontology terms in PPROM affected amnion tissue. Significantly up or downregulated biological process gene ontology (GO) terms which were significantly enriched (FDR < = 0.01) among differentially regulated genes in PPROM affected and unaffected amnion tissue. **B**. Bubble plots of significantly altered cellular components gene ontology terms in PPROM affected amnion tissue. Significantly up or downregulated cellular component gene ontology (GO) terms which were significantly enriched (FDR < = 0.01) among differentially regulated genes in PPROM affected and unaffected amnion tissue. **C**. Bubble plots of significantly altered molecular function gene ontology terms in PPROM affected amnion tissue. Significantly up or downregulated molecular function gene ontology (GO) terms which were significantly enriched (FDR < = 0.01) among differentially regulated genes in PPROM affected and unaffected amnion tissue

A total of 89 GO terms were significantly enriched (FDR < = 0.01) among the DE genes for amnion. Within the Biological Process category, over 60 terms were significantly enriched for upregulated genes, while only the term *extracellular matrix organization* was significantly enriched in downregulated genes (Fig. 3A). In the Cellular Component category, the terms *plasma membrane, extracellular region, and cytoplasm* were among the 15 significantly enriched terms for upregulated genes, while *plasma membrane, extracellular matrix extracellular exosome, and basement membrane* were the only terms significantly enriched in downregulated genes (Fig. 3B). Lastly, in the Molecular Function category, *signal transducer activity, receptor activity, protein binding and collagen binding* were among the terms significantly enriched for the upregulated genes, while only the term *extracellular matrix organization* was significantly enriched for the downregulated genes (Fig. 3C).

### Chorion

**Fig. 4:**
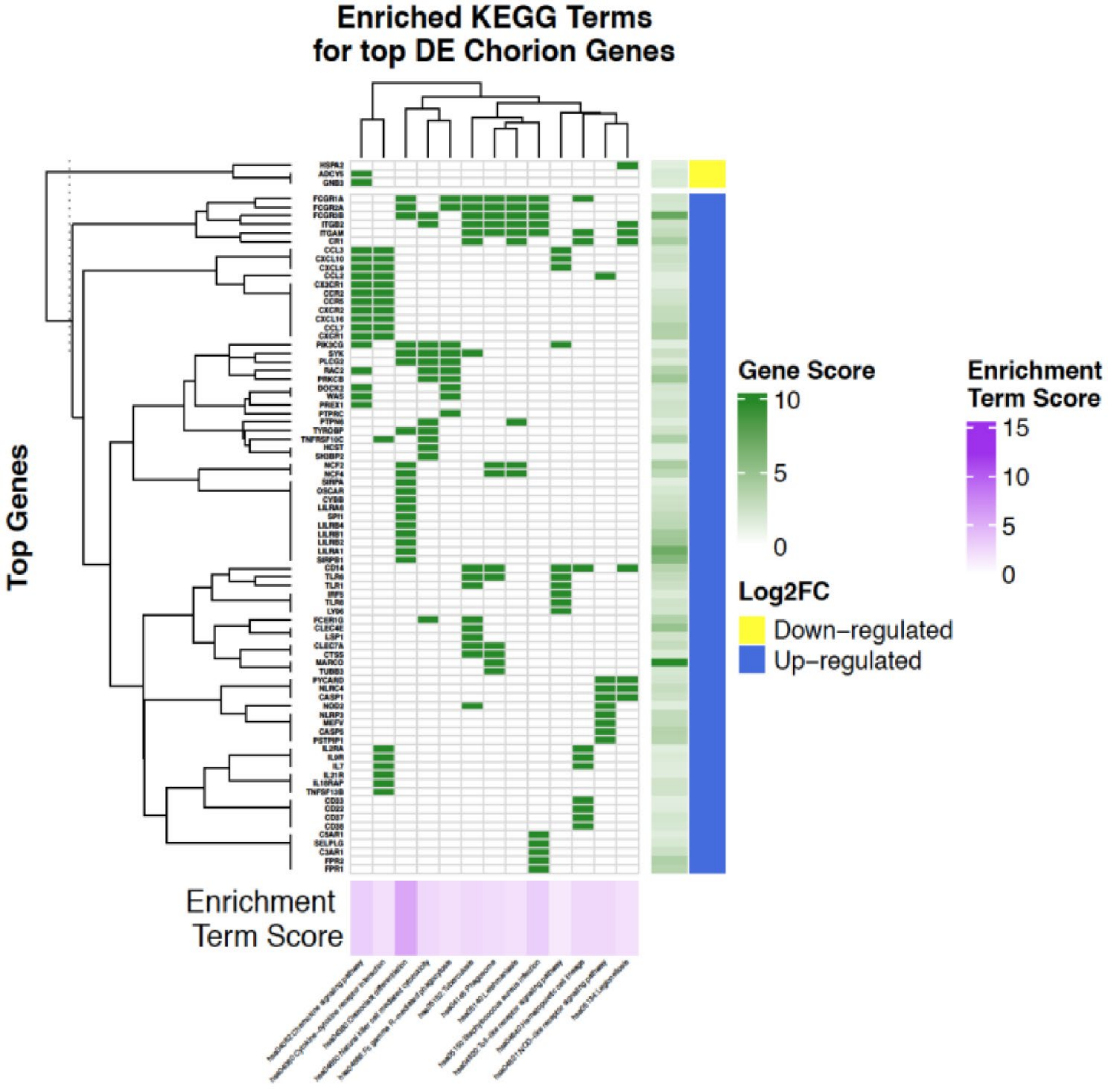
Clustered heatmap of significantly altered KEGG pathways in PPROM affected chorion tissue. Heatmap of differentially expressed genes between PPROM affected and unaffected chorion tissue among significantly enriched Kyoto Encyclopedia of Genes and Genomes (KEGG) pathways clustered by row and column means. The -log10(adjusted p-value) derived from differential expression analysis is illustrated by the Gene Score color bar. The false discovery rate (FDR)-corrected p-value derived from functional enrichment analysis using the DAVID Functional Annotation Tool is illustrated by the Enrichment Term Score color bar. The Log2FC color bar identifies sets of genes which are significantly up or downregulated. The significantly enriched KEGG pathways (FDR p-value < = 0.05) in which the differentially expressed genes are a member of are labeled on the lower axis.

In the chorion, the most significantly enriched (FDR < 0.01) KEGG pathways in upregulated genes included chemokine signaling pathway, NOD-like receptor signaling pathway, cytokine-cytokine receptor interactions pathway, and Toll-like receptor signaling pathway (Fig. 4). There were no significantly enriched (FDR < 0.01) KEGG pathways for downregulated genes in the chorion (Fig. 4).

**Fig. 5:**
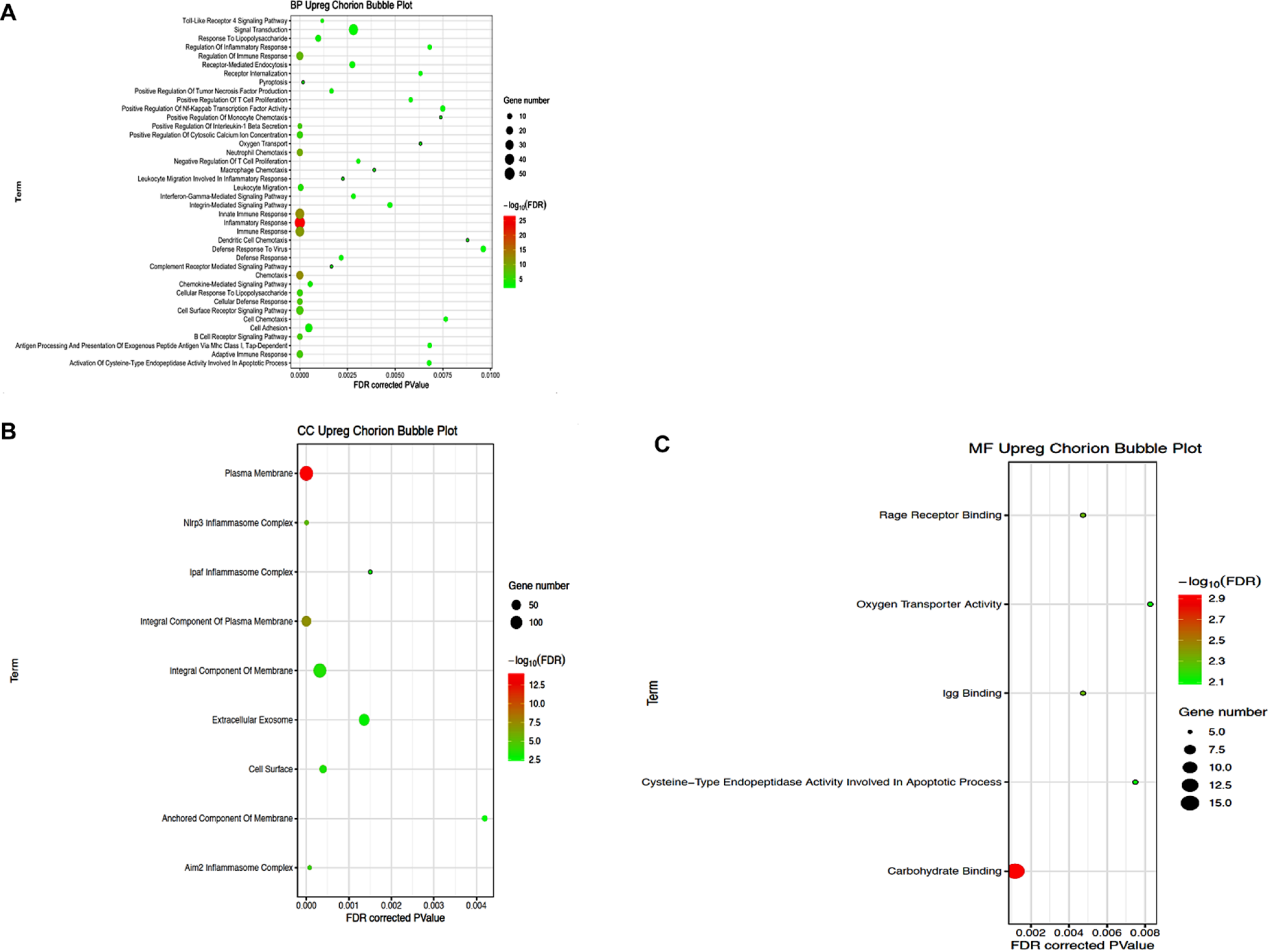
**A**. Bubble plots of significantly altered biological process gene ontology terms in PPROM affected chorion tissue. Significantly upregulated biological process gene ontology (GO) terms which were significantly enriched (FDR < = 0.01) among differentially regulated genes in PPROM affected and unaffected chorion tissue. No downregulated terms were significantly enriched (FDR < = 0.01). **B**. Bubble plots of significantly altered cellular components gene ontology terms in PPROM affected chorion tissue. Significantly upregulated cellular component gene ontology (GO) terms which were significantly enriched (FDR < = 0.01) among differentially regulated genes in PPROM affected and unaffected chorion tissue. No downregulated terms were significantly enriched (FDR < = 0.01). **C**. Bubble plots of significantly altered molecular function gene ontology terms in PPROM affected chorion tissue. Significantly upregulated molecular function gene ontology (GO) terms which were significantly enriched (FDR < = 0.01) among differentially regulated genes in PPROM affected and unaffected chorion tissue. No downregulated terms were significantly enriched (FDR < = 0.01)

A total of 54 GO terms were significantly enriched (FDR < = 0.01) among the DE genes for chorion. Within the Biological Process category, 40 terms were significantly enriched for upregulated genes *including cell adhesion, signal transduction and inflammatory response*, while no terms were significantly enriched in downregulated genes (Fig. 5A). In the Cellular Component category, the terms *plasma membrane, integral component of plasma membrane, and extracellular exosome* were among the 9 significantly enriched terms for upregulated genes, while no terms were significantly enriched in downregulated genes (Fig. 5B). Lastly, in the Molecular Function category 5 terms were significantly enriched for upregulated genes, including *rage receptor binding, carbohydrate binding, and oxygen transporter activity*, while no terms were significantly enriched for downregulated genes (Fig. 5C).

### Validation of gene expression by RT-PCR

To validate genes that were observed to be significantly upregulated in chorionic tissue of PPROM births during RNA-Seq analysis, the expression of 6 genes was assessed by PCR– S100A8, GBP5, MMP25, CXCL9, ALPL, and CASP5. All 6 genes showed upregulation between PPROM cases and controls, although the difference between the two groups did not reach statistical significance for the genes evaluated.

## Discussion

Preterm birth is a major cause of morbidity and mortality in infants, yet minimal progress has been made in recent years to identify the pathophysiological pathways that lead to preterm birth. When studied as a single outcome, it is difficult to identify specific pathways or predictive biomarkers for preterm birth because it is a multifactorial syndrome. Recent studies of the transcriptome of gestational tissue have focused on elucidating the pathways involved in preterm birth by comparing tissue from term deliveries with tissue from preterm deliveries [22–24]. This approach leads to the comparison of DE genes from healthy control (term) tissue to pathological preterm tissue, which introduces a variability in the gestational age of the tissues being compared. Thus, by approaching preterm birth as the functional outcome of either PTL or PPROM, the gestational age variable is removed, and a more vigorous comparison of DE genes can occur. Our study is the first to report transcriptomic data for preterm birth amnion and chorionfrom PTL + PPROM cases compared to PTL only controls. We are also the first to separately analyze the fetal membrane layers to obtain a more complete profile of the fetal membrane transcriptome.

The amnion layer of fetal membranes is composed of collagen fibrils, amnionic cells, and the non-cellular scaffolding ECM in which cells are embedded; thus, the amnionic tissue is believed to correlate with fetal membrane structure and integrity. Our KEGG pathways analysis of amnionic tissue revealed the ECM-receptor pathway to be the most significantly enriched among downregulated genes. Gene ontology term analysis also indicated ECM organization and ECM matrix pathways were enriched among down-regulated genes. Many of these pathways are comprised of multiple genes from the COL family. The downregulation of collagen genes may alter the composition ratio of collagen content in the amnion, which is known to affect tissue rigidity [25, 26]. This multi-pathway down-regulation reported in our study corresponds with previous reviews of the significant role ECM structure and metabolism plays in the pathophysiology of PPROM [27, 28].

The chorion layer of fetal membranes serves as a barrier to inflammatory, immune, and microbial invasion. In our study, the most significantly enriched KEGG pathways in up-regulated genes in the chorion revealed chemokine, NOD receptor, Toll-like receptor, and cytokine-cytokine receptor signaling pathways to be the most significantly enriched. Similarly, KEGG pathway enrichment analysis for upregulated genes in the amnion included three inflammatory pathways: cytokine-cytokine interaction, TNF signaling and the CXCL family. These findings are consistent with previous studies which have described dysregulation of cytokines and chemokines to be involved in PPROM [29–31], and confirm that both layers of the fetal membrane are targets of inflammatory pathway dysregulation during PPROM. Interestingly, neither KEGG pathways nor gene ontology enrichment analyses identified any pathways enriched for down-regulated genes in the chorion.

Fetal membrane weakening is a physiological process that occurs at term, but when dysregulated, can result in PPROM. Inflammation is an essential component of this process, with sterile and septic inflammation considered involved in PPROM. Several markers involved with inflammation have been reported to significantly increase in maternal blood, amnionic fluid, and fetal membrane in cases of PPROM [10, 11, 32, 33]. In our study, GBP5 was significantly upregulated in chorionic tissue from PTL + PPROM births compared to PTL only births. Guanylate-binding proteins (GBPs) are a family of GTPases that play an essential role in cell-autonomous immunity. Studies have also indicated that GPBs have essential functions in controlling inflammation and innate immunity through the activation of inflammasome complexes, with GBP5 promoting NLRP3 inflammasome assembly and immunity [34–36]. Inflammasomes have been shown to be important to host defense mechanisms in pathophysiological inflammatory processes in the chorioamniotic membranes that accompany labor [37].

CXCL9, a major chemokine that controls the migratory and recruitment patterns of T helper 1 cells, is thought to contribute to proinflammatory responses required for labor onset [38, 39]. Serum CXCL9 levels have been reported to be significantly lower in pregnancies complicated by PPROM when compared to normal pregnancies [40]. Fetal membrane CXCL9 expression has been reported to be significantly increased in patients with PTL compared to term labor [39]. Our study is the first to show that CXCL9 is significantly upregulated in the chorion layer of fetal membranes from patients with PTL + PPROM, versus only PTL.

ALPL, the gene that encodes the alkaline phosphatase enzyme, was also significantly upregulated in chorionic tissue from PPROM cases. Previously, studies have shown that serum ALPL rises in normal pregnancies resulting from an increased production of ALPL by the placenta, and it has often been suggested as a potential serum biomarker for preterm birth [41, 42]. Recently, a study by Paquette et al. determined that ALPL is strongly decreased in preterm birth placenta, but significantly increased in the chorioamniotic membranes in preterm birth cases [43]. Our study confirms the significant increase in ALPL in the chorioamniotic membranes, and further specifies the preterm birth pathophysiology to be PPROM.

S100A8, a low molecular weight proinflammatory protein, was significantly upregulated in the chorion of patients with PTL + PPROM compared to the chorion from patients with only PTL. Significantly elevated serum levels of S100A8 have previously been reported in patients with early pregnancy loss and other adverse pregnancy outcomes [44, 45]. Elevated S100A8 has also been reported in the uterine decidua of patients with recurrent early pregnancy loss [46].

MMP25 is a glycosyl-phosphatidylinositol protease, which degrades ECM proteins, including type 4 collagen, gelatin, fibronectin, and fibrin [47]. Fortunato and Menon have shown that human fetal membranes from PPROM pregnancies have fully functioning MMP systems, with several MMPs expressed in the amniochorion during PPROM, but not MMP25 [48, 49]. Our study showed MMP25 to have the most significant upregulation in chorionic tissue of PPROM pregnancies than any of the other MMPs in the transcriptomic profile. The difference in reported results may be based in the fact that the fetal membrane samples from their study were from PPROM patients not in labor, whereas our fetal membrane samples were from PPROM patients with PTL. Additionally, their studies extracted RNA from intact chorioamniotic tissue, whereas we measured RNA from the amnion and chorion separately.

A caveat to the findings described in this study is the small sample size used in the study. Since our study only collected tissue from preterm births, this limited the number of eligible births to 10% of the total births. In a normally distributed population, PPROM is the cause of 1/3 of preterm births, thus decreasing the occurrence even further. To minimize the potential for confounding variables in this small sample size, we carefully matched the samples by gestational age and mode of delivery. Additionally we were unable to extract sufficient amounts of RNA from the 4 cases and 4 control samples to perform both RNA-Seq and RT-PCR on each sample. We chose to run RNA-Seq on the gestationally age-matched and delivery mode-matched samples and run the RT-PCR controls with the same mean gestational age (33w2d), but not delivery-mode matched. Lastly, it would be informative to perform immunohistochemical localization on the fetal membranes to provide further insight into the contribution of cellular heterogeneity to the transcriptomic differences presented in our study.

## Conclusion

In our study, transcriptome analysis of preterm fetal membranes revealed previously reported as well as distinct DE pathways and genes for PPROM, separate from PTL. We are the first to report transcriptome data that reflects the individual pathophysiology of amnion and chorion tissue in PPROM. Although the fetal membrane sample was small, we are certain that we have presented riveting data to support our hypothesis that PPROM and PTL are two separate pathologies of preterm birth. These findings will need to be verified in a larger sample of fetal membranes.

### Electronic supplementary material

Below is the link to the electronic supplementary material.

Supplementary Material 1

Supplementary Material 2

Supplementary Material 3

Supplementary Material 4

## Data Availability

The data sets generated and/or analyzed during the current study are available in the GEO repository and are accessible through GEO series accession number GSE243831. https://www.ncbi.nlm.nih.gov/geo/query/acc.cgi?acc=GSE243831.

## Abbreviations

ALPL: alkaline phosphatase, biomineralization associated
CASP5: caspase 5
CXCL9: c-x-c motif chemokine ligand 9
DE: differentially expressed
DNB: DNA nanoball
ECM: extracellular matrix
FDR: false discovery rate
GBP5: guanylate binding protein 5
GO: gene ontology
S100A8: S100 calcium binding protein A8
MMP25: matrix metallopeptidase 25
PCA: principal component analysis
PPROM: preterm prelabor rupture of membranes
PTL: preterm labor
RIN: RNA integrity number
RNA-Seq: RNA sequencing
RT-PCR: ssCIR DNA:single strand circular DNA
